# The capability set for work – correlates of sustainable employability in workers with multiple sclerosis

**DOI:** 10.1186/s12955-018-0942-7

**Published:** 2018-06-01

**Authors:** D. A. M. van Gorp, J. J. L. van der Klink, F. I. Abma, P. J. Jongen, I. van Lieshout, E. P. J. Arnoldus, E. A. C. Beenakker, H. M. Bos, J. J. J. van Eijk, J. Fermont, S. T. F. M. Frequin, K. de Gans, G. J. D. Hengstman, R. M. M. Hupperts, J. P. Mostert, P. H. M. Pop, W. I. M. Verhagen, D. Zemel, M. A. P. Heerings, M. F. Reneman, H. A. M. Middelkoop, L. H. Visser, K. van der Hiele

**Affiliations:** 1 National Multiple Sclerosis Foundation, Mathenesserlaan 378, Rotterdam, 3023 HB The Netherlands; 2 Department of Psychology, Section Health, Medical and Neuropsychology, Leiden University, PO Box 9555, Leiden, 2300 RB The Netherlands; 3 Department of Neurology, Elisabeth-TweeSteden Hospital, PO Box 90151, Tilburg, 5000 LC The Netherlands; 4 Department of Care Ethics, University of Humanistic Studies, PO Box 797, Utrecht, 3500 AT The Netherlands; 5 Tilburg School of Social and Behavioural Sciences, Tranzo Scientific Centre for Care and Welfare, Tilburg University, PO Box 90153, Tilburg, 5000 LE The Netherlands; 6 Department of Community & Occupational Medicine, University of Groningen, University Medical Centre Groningen, PO Box 30001, Groningen, 9700 RB The Netherlands; 7 MS4 Research Institute, Ubbergseweg 34, Nijmegen, 9522 KJ The Netherlands; 8 van Lieshout Arbo Advies, PO Box 325, Uden, 5400 AH The Netherlands; 9 Department of Neurology, Medical Centre Leeuwarden, PO Box 888, Leeuwarden, 8901 BR, The Netherlands; 10 Department of Neurology, St. Anna Hospital, PO Box 90, Geldrop, 5660 AB The Netherlands; 11 Department of Neurology, Jeroen Bosch Hospital, PO Box 90153, s-Hertogenbosch, 2000 ME The Netherlands; 12 Department of Neurology, Amphia Hospital, PO Box 90158, Breda, 4800 RK The Netherlands; 13 Department of Neurology, St. Antonius Hospital, PO Box 2500, Nieuwegein, 3430 EM The Netherlands; 14 Department of Neurology, Groene Hart Hospital, PO Box 1098, Gouda, 2800 BB The Netherlands; 15 Department of Neurology, Catharina Hospital, PO Box 1350, Eindhoven, 5602 ZA The Netherlands; 16 Department of Neurology, Zuyderland Medical Centre, PO Box 5500, Sittard, 6130 MB The Netherlands; 17 Department of Neurology, Rijnstate Hospital, PO Box 9555, Arnhem, 6800 TA The Netherlands; 18 Department of Neurology, VieCuri Medical Centre, PO Box 1926, Venlo, 5900 BX The Netherlands; 19 Department of Neurology, Canisius-Wilhelmina Hospital, PO Box 9015, Nijmegen, 6500 GS The Netherlands; 20 Department of Neurology, Albert Schweitzer Hospital, PO Box 444, Dordrecht, 3300 AK the Netherlands; 21 Department of Rehabilitation Medicine, University Medical Centre Groningen, University of Groningen, PO Box 30.002, Haren, 9750 RA the Netherlands; 22 Department of Neurology, Leiden University Medical Centre, PO Box 9600, Leiden, 2300 RC The Netherlands

**Keywords:** Multiple sclerosis, Work, Sustainable employability, Capability set for work, Health

## Abstract

**Background:**

The aim of this study was to examine whether work capabilities differ between workers with Multiple Sclerosis (MS) and workers from the general population. The second aim was to investigate whether the capability set was related to work and health outcomes.

**Methods:**

A total of 163 workers with MS from the MS@Work study and 163 workers from the general population were matched for gender, age, educational level and working hours. All participants completed online questionnaires on demographics, health and work functioning. The Capability Set for Work Questionnaire was used to explore whether a set of seven work values is considered valuable (A), is enabled in the work context (B), and can be achieved by the individual (C). When all three criteria are met a work value can be considered part of the individual’s ‘capability set’.

**Results:**

Group differences and relationships with work and health outcomes were examined. Despite lower physical work functioning (U = 4250, *p* = 0.001), lower work ability (U = 10591, *p* = 0.006) and worse self-reported health (U = 9091, *p* ≤ 0.001) workers with MS had a larger capability set (U = 9649, p ≤ 0.001) than the general population. In workers with MS, a larger capability set was associated with better flexible work functioning (*r* = 0.30), work ability (*r* = 0.25), self-rated health (r = 0.25); and with less absenteeism (*r* = − 0.26), presenteeism (*r* = − 0.31), cognitive/neuropsychiatric impairment (*r* = − 0.35), depression (*r* = − 0.43), anxiety (r = − 0.31) and fatigue (*r* = − 0.34).

**Conclusions:**

Workers with MS have a larger capability set than workers from the general population. In workers with MS a larger capability set was associated with better work and health outcomes.

**Trial registration:**

This observational study is registered under NL43098.008.12: ‘Voorspellers van arbeidsparticipatie bij mensen met relapsing-remitting Multiple Sclerose’. The study is registered at the Dutch CCMO register (https://www.toetsingonline.nl). This study is approved by the METC Brabant, 12 February 2014. First participants are enrolled 1^st^ of March 2014.

## Background

Work participation is important from both a societal and personal perspective. In many European countries greater and prolonged work participation is necessary to meet the economic and social demands of an aging society [1, 2]. This means that it is important to invest in keeping both ageing and disabled workers engaged with the labour market. From a personal perspective, work offers not only financial benefits, but also the ability to use knowledge and skills and to have meaningful contacts with others [3, 4].

Multiple Sclerosis (MS) is a chronic disorder of the central nervous system, often diagnosed in young or middle adulthood. MS is characterized by a wide range of symptoms, including disturbances in motor, visual, sensory, autonomic systems and fatigue [5]. Work participation is compromised when an individual has MS. Previous research showed that work participation decreased from 75 to 80% in the early stages of the disease (Expanded Disability Status Scale (EDSS): 0–1) to less than 5% in the very late stages of the disease (EDSS: 7–9) [6]. Job sustainability was found to be influenced by many different factors, including MS symptoms, financial and personal considerations, attitudes towards work and the workplace environment [7]. Personal considerations and attitudes towards work that stimulate job retention included, among others, ‘being interested in your work’, ‘being motivated’, ‘social interaction’ and ‘certainty about your capabilities’ [7]. The complexity of the costs and benefits of work must be taken into account as a balance between work and other aspects in life is of great importance [8]. It should be noted that life outside work is also an important determinant of sustainable employability [9].

It seems that in order to stimulate work participation in patients with MS, people should perceive their daily work as a valuable part of life and not as a burden.

Van der Klink et al. [10] recently developed a model of sustainable employability based on Sen’s capability approach [11]. The following definition of sustainable employability was formulated; ‘sustainable employability means that throughout their working lives, workers can realize tangible opportunities in the form of a set of capabilities. They also enjoy the necessary conditions that allow them to make a valuable contribution through their work, now and in the future, while safeguarding their health and welfare. This requires on the one hand a work context that facilitates them, and on the other hand the attitude and motivation to exploit these opportunities’. In this definition a set of capabilities refers to valued aspects of work that workers are both enabled and able to achieve.

Theoretically, having a larger capability set is linked to better quality of working life and the achievement of valuable functioning, e.g. better work ability, engagement and performance. Consequently, seven work values were identified: (i) use of knowledge and skills, (ii) development of knowledge and skills, (iii) involvement in important decisions, (iv) building and maintaining meaningful contacts at work, (v) setting your own goals, (vi) having a good income and (vii) contributing to something valuable [3]. A new instrument was developed based on Van der Klink’s model of sustainable employability, the ‘Capability Set for Work Questionnaire’. This questionnaire represents an operationalization of the ‘capability set’ [3, 10]. The questionnaire explores whether the set of seven work values are considered valuable by the worker (A), are enabled in the work context (B), and can be achieved (C). An individual work value is considered part of the ‘capability set’ of an individual worker when it is considered important (A), and the workplace offers the opportunity to achieve the value (B), and the worker is able to achieve the value (C). A larger capability set was associated with better work functioning in a general working population [3].

Employment research in MS has had a large focus on demographic and disease-related factors while little evidence exists on the influence of the work context [12] and one’s personal view of the importance of work and what one perceives as valued aspects of work. Research on the mutual influence of MS and the conditions necessary for sustainable employability may help to better understand how to create an optimal situation so that workers with MS remain engaged in the labour market.

The first aim of the current study was to examine whether the capability set and work values of workers with MS differed from matched peers in terms of importance, enablement and achievement. Additionally, discrepancies between importance, opportunities and achievement within the individual work values were examined exploratively. The second aim was to investigate whether the capability set was related to work-related and health outcomes. We expected that due to compromised health in workers with MS, work values cannot be achieved as easily as in workers from the general population, which in turn would lead to a smaller capability set in workers with MS. Additionally, we expected to find conflicts between the importance, opportunities and actual achievement of work values in workers with MS. Furthermore, we expected to find relations between a larger capability set and better work and health outcomes.

## Methods

### Design

A cross-sectional study design was used.

### Participants

Three hundred eight patients with MS were recruited in the context of the MS@Work study via MS outpatient clinics in the Netherlands [13]. Inclusion criteria for the current study were (a) a diagnosis of relapsing-remitting MS (RRMS) according to the Polman-McDonald criteria 2010 [14], (b) 18 years and older, (c) currently having a paid job (d) completing the ‘Capability Set for Work Questionnaire’. Patients with co-morbid psychiatric or neurological disorders, substance abuse, neurological impairment that might interfere with cognitive testing or unable to speak and/or read Dutch were excluded from the study. Five patients were excluded because of an unclear diagnosis. A total of 72 patients with MS did not have a paid job. Another 68 workers with MS did not receive the Capability Set for Work Questionnaire, because the questionnaire was added at a latter point in time to the MS@Work study. This led to the inclusion of 163 workers with MS (77% females; median age 43.0, ranging from 24 to 64 years old). The group of 68 workers with MS who were excluded due to missing Capability Set for Work Questionnaire-data did not differ in terms of age, gender, educational level, disability level, disease duration and work hours.

All workers with MS completed online questionnaires on work functioning and MS symptoms, and underwent neuropsychological and neurological examinations (methods and data to be reported elsewhere). We used data from a large survey study meant to evaluate the construct validity of the Capability Set for Work Questionnaire [3]. The validation study used a panel agency to approach a representative sample (*N* = 1250) of the Dutch general working population. In the current study we included 163 Dutch workers matched for gender, age, level of education and working hours. More details about the survey study can be found in Abma et al. [3].

### Ethical approval

The MS@Work study was approved by the Medical Ethical Committee Brabant (NL43098.008.12 1307) and the Board of Directors of the participating MS outpatient clinics. All subjects provided written informed consent. For the panel study no ethical approval was necessary according to the medical ethics committee of the University Medical Centre Groningen as it did not qualify for being tested according to the Dutch Medical Research Involving Human Subjects act of 1998 [15].

The panel study was performed according to the guidelines of the Association of Universities in the Netherlands [16]. Participants provided online informed consent [3]. Both studies were performed in agreement with the Declaration of Helsinki [17].

### Measures

*Work capabilities* were examined using the Capability Set for Work Questionnaire (CSWQ) in both groups. This questionnaire represents an operationalization of the ‘capability set’ derived from the model of sustainable employability [3, 10]. The questionnaire explores whether a set of seven valued work aspects, are considered valuable by the worker (A), are enabled in the work context (B), and can be achieved (C). In relation to each of these seven valued work aspects the worker is asked (A) ‘How important is <the value> for you?’ (B) ‘Does your work offer the opportunities to achieve <the value>’ and (C) ‘To what extent do you actually achieve <the value>?’ on a scale from 0 = ‘definitely not’ to 5 = ‘very much’. An individual value is considered part of the capability set of an individual worker when it is considered important (A) (score 4–5), and the workplace offers the opportunity to achieve the value (B) (score 4–5), and the worker is able to achieve the value (C) (score 4–5). The capability set can therefore encompass up to seven values. An overall question for the capability for work was posed: ‘Taking all things together, I think I have enough opportunities to remain working’, which required a response ranging from 1 = ‘totally disagree’ to 5 = ‘totally agree’.

*Education level* was in both groups classified based on the Dutch classification system, according to Verhage et al. [18]. Education level was divided into three levels; low, middle or high education. People were considered to have a low level of education up to finishing low level secondary school. Middle education corresponds with finishing secondary school at a medium level. People were considered highly educated when they finished secondary school at the highest level and/or obtained a college or university degree.

*Work ability* was in both groups examined using the item ‘current work ability compared with the lifetime best’ of the Work Ability Index (WAI) [19]. Possible scores range from 0 = ‘completely unable to work’ to 10 = ‘work ability at its best’. The use of a single item of the WAI has been shown to be valid and simple indicator of work ability [20].

*Work functioning* was examined in both groups with two subscales from the Work Role Functioning Questionnaire 2.0 (Dutch Version) (WRFQ 2.0) [21]: physical and flexibility demands. The WRFQ 2.0 measures the perceived percentage of time that physical and emotional problems impact certain work demands. Scores range from 0 to 100, with higher scores indicating better work functioning.

*Self-rated health* was measured in both groups with a question from the Short Form-12 [22]: ‘In general, how would you rate your health?’. Response categories ranged from 1 (very good) to 5 (very poor), which were recoded in a way that a higher score represent a better self-rated health.

*Working hours* was measured in hours per week in both groups.

*Absenteeism* was measured using number of days absent from sickness in the past 3 months (in the general working population) or the number of days absent from work in the past year (in workers with MS). In workers with MS absenteeism was measured on an ordinal scale. 0 days, 1–3 days, 4–5 days, 6–10 days, 11–20 days, 20 days – 6 weeks, 7–13 weeks, 3–6 months, 6 months- 1 year, or not applicable. It was not possible to compare this variable between groups, due to the use of different scales. We did look at correlations between the capability set and the separate measures of absenteeism.

*Presenteeism* was measured only in workers with MS and represents the self-reported influence of MS symptoms on work productivity on a scale from 1 to 10 in which higher scores represent more influence of the MS symptoms on work productivity. This question is part of the Work Productivity and Activity Impairment Questionnaire [23].

*Disability level* in workers with MS was quantified using the Expanded Disability Status Scale (EDSS) [24]. Scores range from 0 (normal neurological exam) to 10 (death due to MS) and increment with steps of 0.5. The EDSS was assessed by a neurologist during the neurological examination (as part of the MS@Work study) at the outpatient clinic where the patient with MS is being treated. Scores between 0 and 3.5 represent mild disability, 4.0–6.5 represent moderate disability and scores of 7.0 and above are seen as a severe level of disability [6].

*Cognitive and neuropsychiatric functioning* was measured in workers with MS using the Multiple Sclerosis Neuropsychological Screening Questionnaire (MSNQ) [25]. Scores range from 0 to 60 and higher scores are indicative of greater subjective cognitive and neuropsychiatric impairment.

*Information processing speed* was examined using the Symbol Digit Modalities Test (SDMT) [26] in workers with MS. The SDMT was administered by a trained research nurse or (neuro)psychologist during a cognitive examination (as part of the MS@Work study) at the outpatient clinic where the patient with MS is being treated. The SDMT is often used as an indicator of cognitive functioning in MS [27]. Possible total scores range from 0 to 110. Higher scores indicate better performance. Z-scores were retrieved from the SDMT manual, Z-scores below − 1,5 were considered indicative for an impairment in information processing speed [26].

*Anxiety and depression* were examined in workers with MS using the Hospital Anxiety and Depression Scale (HADS) [28]. Possible scores per domain, i.e. anxiety or depression, range from 0 to 21 and scores at or above 8 are considered indicative of major depression or generalized anxiety disorder as validated in MS [29].

*Fatigue impact* was measured in workers with MS using the Modified Fatigue Impact Scale (MFIS) [30]. This scale assesses the impact of fatigue on daily functioning in physical, cognitive, and psychosocial dimensions. Possible total scores range from 0 to 84. Total scores at or above 38 are considered indicative of MS-related fatigue [31].

### Statistical analysis

SPSS for Windows (release 23.0) was used for data analysis. Workers from the general population were matched with workers with MS using fuzzy case-control matching for gender (tolerance = 0), age (tolerance = 8), working hours (tolerance = 5) and level of education (tolerance = 1). Differences in demographics, self-rated health, work ability, work functioning and capability aspects between workers with MS and the general working population were analysed using parametric or non-parametric tests. Additionally, using Wilcoxon Signed Rank Tests, we examined discrepancies between the importance of each value (A), whether the value was enabled (B) and whether the person was able to achieve the value (C), but only for important values (score 4–5). Spearman’s rho correlation analyses were performed to examine whether the capability set and overall capability item were related to measures of work functioning and health. Correlation coefficients between 0.15–0.29 were interpreted as weak, 0.3–0.59 were interpreted as moderate and 0.6–1 were interpreted as strong [32]. To correct for multiple testing a Bonferroni correction was used for the interpretation of statistical significance. Due to the exploratory nature of the analyses for discrepancies between A, B and C, a *p* value of 0.01 was considered trend significant.

## Results

### Demographics, work and self-reported health

The MS sample and the matched group included especially females (77%) in their early 40’s (see Table 1). The majority of workers with MS were employed in office and administrative support (22%), the healthcare sector (15%), education, training and library (9%) and business and financial services (8%). The majority of workers from the general population were employed in the healthcare sector (26%), education (7%), the retail industry (7%) and other business services (7%) (note; in 17% type of work was reported as ‘other’ and not further specified). The majority of workers with MS reported 1–3 days absent (27%), 0 days (24%) and 4–5 sick days (16%) in the past year. In the general population the median days called in sick was 0 (IQR = 0), with 76.5% of the general population reporting no absenteeism in the past 3 months.

**Table 1:** Demographic, self-rated health, work ability and work functioning findings in workers with MS and workers from the general population

	Workers with MS (*N* = 163)					General working population (*N* = 163)					Test statistics		
	%	Mean	Median	IQR	Min - max	%	Mean	Median	IQR	Min-Max	X^2^	U	*p*
Gender (female) (in %)	77.3%					76.7%					0.17		0.89
Age		42.5	43.0	15.0	24–64		42.5	44.0	16.0	19–67		13163.0	0.89
Educational level											3.07		0.22
Low (%)	14.1%					14.7%							
Medium (%)	41.7%					50.3%							
High (%)	44.2%					35.0%							
Self-rated health (scale 1–5)^a^		2.9	3.0	1.0	1–5		3.4	3.0	1.0	2–5		9091.0	< 0.001^*^
Number of work hours per week		29.1	30.0	17.0	6–60		29.9	30.0	16.0	5–60		12520.5	0.37
Work ability (scale 0–10)^a^		7.1	8.0	2.0	0–10		7.7	8.0	2.0	0–10		10590.5	0.006^*^
Work Functioning Physical (scale 0–100)^a^		77.4	90.0	35.6	0–100		89.9	100.0	15.0	25–100		4250.0	0.001^*^
Work Functioning Flexibility (scale 0–100)^a^		75.3	85.0	25.0	0–100		81.3	87.5	20.8	16.7–100		10425.0	0.46

Note: *IQR* Inter Quartile Range. Although all data are non-parametric, mean scores are displayed to further clarify the distribution of the data

^a^Higher scores indicate better self-rated health, work ability, and work functioning

^*^Bonferroni corrected *p* values ≤ 0.006 were considered significant

The data on demographics, self-rated health, work ability and work functioning in workers with MS and workers from the general population are presented in Table 1.

There were no group differences in gender, age, educational level, number of work hours or work functioning in terms of flexibility. In both groups, males worked significantly more hours than females. Work hours were equally distributed based on educational level, and age within and between both groups. Workers with MS rated their health, work ability, and physical work functioning lower than workers from the general population (Table 1). Disease characteristics of workers with MS are shown in Table 2. A total of 91.5% of the workers with MS had a mild disability level (EDSS:0–3.5) and 8.5% had a moderate disability level (EDSS:4.0–6.5). A total of 73% of the workers with MS received Disease Modifying Treatment (DMT). Based on the scores on the SDMT 2.5% of the workers with MS had an impairment in information processing speed. Within workers with MS, 11.7% reported scores indicative for a major depression, and 25.2% reported scores indicative for a general anxiety disorder. Scores indicating the presence of MS-related fatigue were reported by 42.3% of the workers with MS.

**Table 2:** Disease characteristics of workers with MS

	Workers with MS (*N* = 163)				
	Median	IQR	Mean	SD	Min-Max
Disability level (1–10)	2.0	1.0			0–6
Disease duration (in years)	6.0	10.0			0–31
Depression (0–21)	2.0	4.0			0–14
Anxiety (0–21)	5.0	5.0			0–21
Fatigue Impact (0–84)			33.7	15.4	0–73
Cognitive and neuropsychiatric functioning (0–61)	19.0	12.0			1–51
Information processing speed (0–110)			53.9	8.3	31–75

Note: *IQR* Inter Quartile Range, *SD* Standard Deviation

In the group of workers from the general population 26.4% reported having a chronic disease, ranging from problems with the human musculoskeletal system (17.2%; e.g. rheumatoid arthritis) to respiratory tract disorders (8.0%; e.g. Chronic Obstructive Pulmonary Disease).

### Work capabilities

A comparison of work capabilities in workers with MS and workers from the general population is presented in Table 3. Many work values were rated as more important (A), more enabled in the work context (B) and more able to achieve (C) by workers with MS than workers from the general working population. Overall, workers with MS had a larger capability set, and had a greater belief in sufficient opportunities to continue working as measured with the overall item of the CSWQ. Within each group, the value ‘use of knowledge and skills’ (in 85 and 67% respectively) and ‘building and maintaining meaningful contacts at work’ (in 82 and 62% respectively) were most often included in the capability set. The work values ‘use of knowledge and skills’, ‘involvement in important decisions’, ‘having a good income’ and ‘contributing to something value’ were more often included in the capability set by workers with MS than in the capability set of workers from the general population.

**Table 3:** Comparison of work capabilities between workers with MS and workers from the general population

	Workers with MS (*N* = 163)					General working population (*N* = 163)					Test statistics		
Work capabilities	%	Mean	Median	IQR	Min-max	%	Mean	Median	IQR	Min-max	X^2^	U	*p*
i. Use of knowledge and skills in your work													
A. Importance		4.6	5.0	1	3–5		4.3	4.0	1	2–5		9823.0	< 0.001^*^
B. Opportunities		4.4	4.0	1	2–5		3.8	4.0	0	1–5		22062.0	< 0.001^*^
C. Able to achieve		4.3	4.0	1	1–5		3.9	4.0	1	1–5		23190.0	< 0.001^*^
Included in capability set (in %)	85%					67%					14.9		< 0.001^*^
ii. Development of knowledge and skills in your work													
A. Importance		4.2	4.0	1	1–5		4.1	4.0	1	1–5		11637.5	0.42
B. Opportunities		3.9	4.0	0	1–5		3.5	4.0	1	1–5		9802.5	< 0.001^*^
C. Able to achieve		3.7	4.0	1	1–5		3.4	4.0	1	1–5		11511.0	0.33
Included in capability set (in %)	57%					49%					2.6		0.11
iii. Involvement in important decisions													
A. Importance		4.3	4.0	1	2–5		3.8	4.0	0	1–5		9066.5	< 0.001^*^
B. Opportunities		3.9	4.0	2	2–5		3.3	3.0	1	1–5		8654.5	< 0.001^*^
C. Able to achieve		3.8	4.0	1	2–5		3.3	3.0	1	1–5		9136.5	< 0.001^*^
Included in capability set (in %)	61%					39%					15.9		< 0.001^*^
iv. Building and maintaining meaningful contacts at work													
A. Importance		4.4	4.0	1	1–5		4.1	4.0	1	2–5		10541.5	< 0.001^*^
B. Opportunities		4.2	4.0	1	1–5		3.9	4.0	0	1–5		10433.0	< 0.001^*^
C. Able to achieve		4.1	4.0	1	1–5		3.8	4.0	1	2–5		10759.0	0.001^*^
Included in capability set (in %)	82%					68%					8.7		0.003
v. Setting your own goals in your work													
A. Importance		4.1	4.0	1	2–5		3.9	4.0	1	1–5		10734.0	0.005
B. Opportunities		3.9	4.0	1	2–5		3.6	4.0	1	1–5		10827.0	0.007
C. Able to achieve		3.8	4.0	1	2–5		3.6	4.0	1	1–5		11152.0	0.03
Included in capability set (in %)	63%					54%					2.5		0.11
vi. Having a good income													
A. Importance		4.1	4.0	1	1–5		4.0	4.0	0	2–5		12105.0	0.41
B. Opportunities		3.9	4.0	1	2–5		3.3	3.0	1	1–5		8276.5	< 0.001^*^
C. Able to achieve		3.8	4.0	1	2–5		3.2	3.0	1	1–5		8092.0	< 0.001^*^
Included in capability set (in %)	62%					39%					16.7		< 0.001^*^
vii. Contributing to something valuable in your work													
A. Importance		4.3	4.0	1	3–5		4.0	4.0	1	1–5		9733.5	< 0.001^*^
B. Opportunities		4.1	4.0	1	2–5		3.6	4.0	1	1–5		8467.5	< 0.001^*^
C. Able to achieve		3.9	4.0	2	2–5		3.6	4.0	1	1–5		9660.0	< 0.001^*^
Included in capability set (in %)	71%					54%					9.1		0.002^*^
Capability set		4.8	5.0	3	0–7		3.7	4.0	4	0–7		9649.0	< 0.001^*^
Overall item: Altogether, I think I have sufficient opportunities to continue to work.		4.3	4.0	1	2–5		3.8	4.0	0	1–5		8476.0	< 0.001^*^

Note: *IQR* Inter Quartile Range. Although all data are non-parametric, mean scores are displayed to further clarify the distribution of the data

^*^Bonferroni corrected *p* values ≤ 0.002 were considered significant

### Differences between the importance (A), being enabled (B) and being able to achieve (C) work values

Within each group we found significant discrepancies between the importance of a value (A) that was considered important (score 4–5) and whether the person was enabled (B) and able to achieve (C) the value (Table 4). In both groups, for every work value the importance (A) was considered higher than being enabled in the work context (B) and being able to achieve (C) the work value.

**Table 4:** Discrepancies between the importance (A), being enabled (B) and being able to achieve (C) work values in workers with MS and workers from the general population

	Workers with MS (*N* = 163)						General working population (*N* = 163)					
Capability aspects	(A-B)^a^		(A-C)^a^		(B-C)^a^		(A-B)^a^		(A-C)^a^		(B-C)^a^	
	Z	*p*	Z	*p*	Z	*p*	Z	*p*	Z	*p*	Z	*p*
i. Use of knowledge and skills in your work	−5.2	< 0.001^**^	− 5.6	< 0.001^**^	− 2.5	0.009^*^	− 6.2	< 0.001^**^	− 6.0	< 0.001^**^	− 0.8	0.40
ii. Development of knowledge and skills in your work	− 5.5	< 0.001^**^	− 7.3	< 0.001^**^	− 4.2	< 0.001^**^	−6.9	< 0.001^**^	− 7.1	< 0.001^**^	− 0.2	0.85
iii. Involvement in important decisions	−6.7	< 0.001^**^	− 7.3	< 0.001^**^	− 2.8	0.005^*^	− 6.7	< 0.001^**^	−7.1	< 0.001^**^	− 0.5	0.59
iv. Building and maintaining meaningful contacts at work	−5.1	< 0.001^**^	− 6.0	< 0.001^**^	−2.6	0.008^*^	−5.3	< 0.001^**^	− 5.8	< 0.001^**^	− 1.4	0.16
v. Setting your own goals in your work	−4.9	< 0.001^**^	−5.7	< 0.001^**^	−2.9	0.004^*^	−5.2	< 0.001^**^	−5.6	< 0.001^**^	−1.7	0.09
vi. Having a good income	−5.6	< 0.001^**^	−5.7	< 0.001^**^	−1.3	0.184	−7.6	< 0.001^**^	−7.8	< 0.001^**^	−1.9	0.05
vii. Contributing to something valuable in your work	−4.6	< 0.001^**^	−6.1	< 0.001^**^	− 4.5	< 0.001^**^	− 6.3	< 0.001^**^	−6.8	< 0.001^**^	−1.2	0.22

Note: Results of the Wilcoxon Signed Rank Test to test discrepancies between importance (A), being enabled (B) and being able to achieve (C) work values are presented

^a^Conflict scores

^**^Bonferroni corrected *p* values ≤ 0.001 were considered significant, ^*^p values ≤ 0.01 were considered trend significant

Discrepancies between being enabled (B) and actually being able to achieve (C) the value were only found within workers with MS. Discrepancies were found in two of the seven values; ‘development of knowledge and skills’ and ‘contributing to something valuable’. In four values trend significant discrepancies were found between being enabled (B) and actually being able to achieve (C). In all these values workers with MS rated the opportunities at work higher than the extent to which they were able to achieve these values.

### Associations between work capabilities, work and health outcomes

Associations between work capabilities and work and health outcomes are presented in Table 5. Weak to moderate associations were found between a larger capability and a larger overall capability item and better work and health outcomes in workers with MS. In the general population a larger capability set and a larger overall capability item were weakly to moderately associated with better work ability.

**Table 5:** Associations between work capabilities, work and health-related outcomes

	Workers with MS (*N* = 163)		General working population (*N* = 163)	
	Capability set	Overall capability item	Capability set	Overall capability item
Age	−0.03	− 0.17	− 0.04	− 0.07
Work hours	0.19	0.29^*^	0.21	0.16
Work ability	0.25^*^	0.43^*^	0.28^*^	0.39^*^
Work Functioning-Physical	0.19	0.39^*^	0.22	0.19
Work Functioning-Flexibility	0.30^*^	0.27^*^	0.11	0.25
Absenteeism	−0.26^*^	−0.29^*^	0.02	−0.02
Presenteeism	−0.31^*^	−0.42^*^	n.a.	n.a.
Self-rated health	0.25^*^	0.39^*^	0.01	0.21
Disease duration	0.12	0.02	n.a.	n.a.
Disability level	−0.05	−0.13	n.a.	n.a.
Cognitive and neuro-psychiatric functioning	−0.35^*^	−0.44^*^	n.a.	n.a.
Information processing speed	0.05	0.09	n.a.	n.a.
Depression	−0.43^*^	−0.39^*^	n.a.	n.a.
Anxiety	−0.31^*^	−0.38^*^	n.a.	n.a.
Fatigue impact	−0.34^*^	−0.44^*^	n.a.	n.a.

Note: Spearman’s rho coefficients are reported. Not all health measures were available for the general working population (*n.a* data not available)

^*^Bonferroni corrected *p* values ≤ 0.001 were considered significant

## Discussion

We observed that workers with MS have a larger capability set than workers from the general population. In both groups we found conflicts between the importance of each work value, and being enabled and actually being able to achieve the work value. Only in workers with MS, a discrepancy between being enabled and actually being able to achieve a work value was found. In workers with MS a larger capability set was weakly to moderately associated with better work and health outcomes.

### Importance of values and opportunities at work

Workers with MS on average rate each work value as important to very important. In fact, workers with MS find it significantly more important than workers from the general population to be able to use knowledge and skills, to be involved in important decisions at work, to build and maintain meaningful contacts at work and to contribute to something valuable.

Having a chronic, inflammatory and neurodegenerative illness like MS may stimulate rethinking and increases awareness of the importance of having a job, and what aspects make work important. This idea fits well in the Shifting Perspectives Model of Chronic Illness, which proposes that a person living with a chronic disease continually goes through shifts in perspective from either ‘illness in the foreground’ to ‘wellness in the foreground’ [33]. When the wellness perspective comes to the foreground, the focus shifts to the self instead of the diseased body, which may lead to a re-appreciation of life and others, including situations (e.g. work) affected by the disease. On the other hand, when the focus shifts to the illness, the world around them may receive less attention. The Shifting Perspectives Model assumes that a person with a chronic disease has one preferred perspective and that no perspective is better or worse than the other. Considering the high importance the workers with MS place on work values, the prevalent perspective in our sample may be ‘wellness in the foreground’. In this respect, we should be aware that our sample may not be generalizable to all workers with MS. Patients with MS who do not find work participation that important, may have left the labour market at an earlier stage. It is possible that the workers with MS in the current study are characterized by a specific MS-phenotype. A selection bias may arise, in that people with more positive personal expectations, a better balance between work and other aspects in life, lower disease severity and more motivation are over represented in our group of workers with MS, causing a possible ‘healthy worker effect phenomenon’ [34]. In addition, it is possible that the participants in the panel study might have placed questions about work and work values into a different perspective. There may have been a higher awareness of these work values in workers with MS, as they know and feel the importance as well as the precariousness of their employment.

Workers with MS generally rate opportunities at work higher than workers from the general population, and feel better able to achieve most values. This is also reflected in a larger capability set and a higher score on the overall capability item in workers with MS compared to workers from the general population. Nevertheless, workers with MS rate their physical work functioning, work ability and self-rated health lower than workers from the general population. It is likely that workers with MS were able to compensate for their lower level of physical work functioning and declining health through an open and supportive climate at work and provision of sufficient opportunities and possible accommodations. Disclosure of disease status [35] may also lead to such an environment. In the current study the vast majority (94%) of the workers with MS have disclosed their disease status to their supervisor. Messmer et al. [7] identified various workplace changes that facilitate work participation for people with MS, including a flexible work schedule, changes in tasks, increased accessibility and time off when needed. In the workers with MS in the current study, 69.9% made some sort of work accommodation, ranging from an accommodation in flexible work scheduling (50.9%; i.e. changed work hours, rest periods), to physical changes to surroundings (41.1%) and cognitive aids (35.6%; i.e. memory aids or written work instructions).

A possible explanation for the increased feeling of being able and enabled to maintain valued aspects of work in workers with MS may lie in the fact that in the current study many workers with MS were able to arrange work accommodations in consultation with their supervisor. This positive experience of being facilitated might change a person’s attitude and sense of control towards work in a positive way [36].

### Conflicts between the importance (A) and being enabled (B) and able to achieve (C) values

In both groups, we found that the importance of valued work aspects, for all seven values, was rated higher than the extent to which the workplace offers opportunities to achieve these values (A-B). Furthermore, the importance of valued work aspects was rated higher than the extent to which the workers were actually able to achieve these values (A-C). Differences between being enabled and actually being able (B-C) to achieve valued work aspects were found within the workers with MS but not in the general population.

The data of the general population used in our study, was part of a larger group of workers from the validation study on the validation of the CSWQ [3], which similarly reported no differences between the B and C items. The assumption of Abma et al. [3] that in workers with differences between B and C, something outside the work situation is hindering workers from succeeding in realizing the work capability, is credible given that these differences were only seen in workers with MS. The adverse health effects of MS, including fatigue, depression, anxiety and subtle decreases in physical functioning, seem to interfere with the possibility to actually achieve these work aspects even though opportunities are offered at work.

### Associations between work capabilities, work functioning and health

Having a larger capability set for work has been associated with better work outcomes in 1157 workers from the general population [3]. The current study corroborates these findings in workers with MS as significant relations were found between a larger capability set and better work outcomes. Moreover, in workers with MS a larger capability set was associated with better health in terms of self-reported cognitive and neuropsychiatric functioning, depression, anxiety and fatigue. Apparently, a larger capability set for work is involved with a more productive and healthier life in workers with MS. Interestingly, the overall capability item ‘altogether, I think I have sufficient opportunities to continue to work’ was also associated with work functioning and cognitive and psychological health in workers with MS, and is additionally correlated with measures of physical work functioning and work hours. This item seems to represent a very broad measure of work functioning and health.

### Strength and limitations

To our knowledge, this is the first study to investigate the ‘value of work’ in workers with MS. The questionnaire used to do so, is validated in one study using workers from the general population [3]. The CSWQ is a fairly new instrument and we should keep in mind that it is important to further investigate its construct validity. In addition, the CSWQ is not specifically validated for people with a chronic illness such as MS. Although we do not have any reason to suggest that the construct of work capabilities is different in people with a chronic illness, we cannot be sure if, how, and to what extend this has introduced bias in this study. In our study the expected associations between the CSWQ with health and work outcomes were replicated. Nonetheless, the CSWQ would benefit from further validation.

A strength of the current study is that we were able to carefully match the workers with MS to workers from the general population based on gender, age, level of education and work hours. The data on workers from the general population included workers with a chronic disease. Although it could be discussed that including workers with a chronic disease introduces a bias, the sample provides a good representation of the general working population of which workers with a chronic disease are also part. Previous research shows that between 25 and 37% of all Dutch workers are affected by a chronic disease [37, 38].

Due to the fact that both study populations are retained from different studies a discrepancy occurred in the manner in which ‘absenteeism’ and ‘job type’ were measured. These measures could not be compared between groups. Moreover, not all health measures that were available for the workers with MS were available for the workers from the general population.

A possible selection bias be present, in a way that only the motivated workers with MS, with mild disease severity were willing to participate in this research. Furthermore, the questionnaires and especially the question on absenteeism may be influenced by a possible recall bias [39, 40]. With the use of questionnaires we should also be aware of the possible response shift bias in both groups, in which cognitive biases shift the response of participants away from an accurate or truthful response [41]. Furthermore, given the cross-sectional nature of this study, causal relationships between health and work outcomes cannot be established.

### Further research

The current study included a group of workers with MS characterized by mild disability and few cognitive problems. Further research could benefit from including a more wide-spread MS phenotype to more thoroughly assess the spectrum of the Capability Set for Work in workers with MS. Moreover, to further explore sustainable employability over time in workers with MS, a longitudinal study is needed to evaluate the predictive validity of the questionnaire. It is of interest to determine what level of discrepancy (A-B) poses a risk for future job loss. On an individual basis, it may then be useful to identify ‘risky’ discrepancies in importance and opportunity of each value, so that workplace opportunities can be adjusted to help workers with MS stay at work.

## Conclusion

In conclusion we found that workers with MS rate work values as more important and have a larger capability set than workers from the general population. A larger capability set was related with better work outcomes in all workers and better health outcomes in workers with MS. Surprisingly, workers with MS felt they were given more opportunities and were actually able to achieve work values better, compared to the general population. The fact that the workplace offers less opportunities to achieve valued work aspects relative to the importance that is given to these aspects, raises concern.

Given the health and productivity benefits of an increased set of work capabilities, such conflict needs to be resolved for individual workers. This process may begin by identifying value conflicts and helping workers and employers to create an optimal task description and a working environment in which important values can be achieved. The CSWQ might be a useful screening tool in this respect.

## Abbreviations

CSWQ: Capability set for work questionnaire
EDSS: Expanded disability status scale
HADS: Hospital anxiety and depression scale
MFIS: Modified fatigue impact scale
MS: Multiple sclerosis
MSNQ: Multiple sclerosis neuropsychological screening questionnaire
RRMS: Relapsing-remitting multiple sclerosis
SDMT: Symbol digit modalities test
WAI: Work ability index
WRFQ 2.0: Work role functioning questionnaire 2.0
